# A novel hierarchical clustering algorithm for gene sequences

**DOI:** 10.1186/1471-2105-13-174

**Published:** 2012-07-23

**Authors:** Dan Wei, Qingshan Jiang, Yanjie Wei, Shengrui Wang

**Affiliations:** 1 Cognitive Science Department & Fujian Key Laboratory of the Brain-like Intelligent Systems, Xiamen University, Xiamen, China; 2 Shenzhen Key Lab for High Performance Data Mining, Shenzhen Institutes of Advanced Technology, Chinese Academy of Sciences, Shenzhen, China; 3 Department of Computer Sciences, University of Sherbrooke, Sherbrooke, QC, Canada

## Abstract

**Background:**

Clustering DNA sequences into functional groups is an important problem in bioinformatics. We propose a new alignment-free algorithm, mBKM, based on a new distance measure, DMk, for clustering gene sequences. This method transforms DNA sequences into the feature vectors which contain the occurrence, location and order relation of *k*-tuples in DNA sequence. Afterwards, a hierarchical procedure is applied to clustering DNA sequences based on the feature vectors.

**Results:**

The proposed distance measure and clustering method are evaluated by clustering functionally related genes and by phylogenetic analysis. This method is also compared with BlastClust, CD-HIT-EST and some others. The experimental results show our method is effective in classifying DNA sequences with similar biological characteristics and in discovering the underlying relationship among the sequences.

**Conclusions:**

We introduced a novel clustering algorithm which is based on a new sequence similarity measure. It is effective in classifying DNA sequences with similar biological characteristics and in discovering the relationship among the sequences.

## Background

With the development of advanced biotechnology, more and more biological sequence information has been generated. The amount of genetic data is growing faster than the rate at which it can be analyzed. Clustering techniques provide a viable solution for handling and analyzing such rapidly growing genetic data. Clustering algorithms partition sequences into different biologically meaningful groups, facilitating therefore the prediction of functions of genes [1]. When a new gene is assigned to a cluster, the biological function of this cluster can be attributed to this gene with high confidence. On the other hand, clustering gene sequences into groups may also help with analyzing evolutionary relationships among the sequences in a cluster [2].

Clustering of gene sequences requires calculation of similarity between sequences. There are two clustering approaches according to the similarity measure used in a clustering method. One is based on sequence alignment. The similarity between two gene sequences is measured by the scores obtained from an alignment algorithm such as BLAST [3] or FASTA [4]. Although sequence alignment gives good solutions, it is relatively difficult to cluster a large number of sequences because of its computational complexity. Moreover, if the sequences in the set vary in length, a satisfactory alignment is hard to achieve, resulting in a low accuracy of clustering.

The other approach for similarity measure is to use alignment-free methods [5-10]. In recent years, several alignment-free measures have been proposed. The word-based measure is one of the most widely used methods [11-14]. This method chooses a short word length *k*, maps each sequence onto an *n*-dimensional vector according to its *k*-length tuple (also called *k*-tuple or *k*-word) properties, and then assesses the similarity of any two vectors by measures such as Euclidean distance [15], Mahalanobis distance [16], Kullback–Leibler discrepancy [17], cosine distance [18] or Pearson’s correlation coefficient [19]. In recent years, several novel alignment-free measures [20,21] have been designed for DNA sequences analysis. Yang et al. [22] extended the *k*-tuple distance, which is based on the difference in tuple frequencies, to clustering gene sequences. Their tuple-based method determines the similarity of sequences by considering only tuple frequencies and ignoring the positional information within a sequence.

Major algorithms used in gene sequence clustering can be divided into two categories according to the result format: hierarchical clustering algorithms and partitional clustering algorithms [23]. Hierarchical clustering is widely used for detecting clusters in genomic data. It generates a set of partitions forming a cluster hierarchy. According to linkage criteria, there are three hierarchical clustering methods including single-linkage clustering (SL), complete-linkage clustering (CL) and average-linkage clustering (AL) [24]. With SL, clusters may be merged together due to single sequences being close to each other, even though many of the sequences in each cluster may be very distant to each other [25]. CL tends to find compact clusters of approximately equal diameters [25]. With CL, all objects in a cluster are similar to each other. AL can be seen as an intermediate between single and complete linkage clustering, resulting in more homogeneous clusters than those obtained by the single-linkage method [26]. For instance, BlastClust [27] and GeneRage [28] employ single linkage clustering approach; SWORDS [29] is based on word frequencies as profiles to merge clusters hierarchically; and Uchiyama [30] use average linkage clustering algorithm to classify genes. Hierarchical approaches may yield fairly good results, but they require the similarity of all pairs of sequences and quickly arrive at a bottleneck in terms of computational time and memory usage for large-scale data sets [31].

Partitioning algorithms have also been used. Partitional clustering obtains a partition of data objects by optimizing some clustering criterion. Partitional clustering algorithms are simple and well-suited for clustering large datasets [32]. K*-*means (KM) [33,34] is a commonly used method of partitional clustering methods. KM has a lower order of computational complexity and demands less physical memory than the hierarchical method. It is suitable for clustering large gene data. Some KM-based algorithms, such as those introduced by Wan et al. [33], Kelarev et al. [34], Tseng et al. [35] and Ashlock et al. [36], have been developed to group DNA sequences. The major drawback of KM compared to hierarchical clustering algorithms is the lack of hierarchical relationships in its results. To remedy the problem, bisecting K-means (BKM), a hierarchical variation of KM, was proposed to build a tree of clusters in a top-down fashion by splitting the least homogeneous cluster into two more homogeneous ones. BKM can produce either a flat clustering or a hierarchical clustering by recursively applying KM. It has a linear complexity and is relatively efficient and scalable. Recent study [37] concluded that BKM outperforms KM and performs equally well or better than hierarchical methods when it partitions the dataset based on a homogeneity criteria. The bisecting approach is very attractive for genomic studies [38].

Hierarchical clustering produces a nested series of partitions, where the results are usually depicted as a dendrogram while partitional clustering produces a flat partition. BlastClust [27] is a hierarchical clustering method based on BLAST scores as the measure of sequence similarity. BlastClust computes pairwise similarity of all sequences by BLAST alignment and then clusters sequences by the single linkage clustering method which produces clusters of linear topology. The performance of BlastClust is limited by the size of the input data. CD-HIT-EST [39], a partitional approach, is also widely used to cluster DNA sequences. CD-HIT-EST uses an incremental clustering process and avoids the unnecessary alignments by a short word filtering mechanism, which detects similar sequences by counting the number of identical short words between them. The purpose of filters is to decide whether the identity between two sequences is above or below a threshold without aligning them, therefore speeding up the clustering process. Though CD-HIT-EST is based on alignment, it can avoid too many pairwise alignments by using a filter, thus it is faster than BlastClust, and can handle larger datasets.

Recent studies reveal also that BlastClust is less effective for clustering divergent sequences [40], and its performance strongly depends on the choice of optimal BLAST parameters including similarity threshold, percent identity, and alignment length [41]. CD-HIT-EST, on the other hand, does not provide hierarchical relationships between clusters of sequences. In many situations both CD-HIT-EST and BlastClust yield clusters with only one sequence [41]. All the traditional clustering methods based on sequence alignment encounter computational difficulties in dealing with large biological databases.

The approach presented in this paper involves a new alignment-free distance measure based on *k*-tuples, DMk (Distance Measure based on *k*-tuples) [42], and a modified bisecting K-means clustering algorithm, mBKM (modified Bisecting K-Means algorithm). mBKM aims to speed up the clustering process by using the alignment-free similarity measure, and is able to produce either a hierarchical clustering or a partition clustering result. We have applied mBKM with DMk in clustering gene sequences and performing phylogenetic analysis. DMk shows better performance than the *k*-tuple distance in our experiments, and mBKM outperforms SL, CL, AL, BKM and KM when tested on public gene sequence datasets. Furthermore, the proposed method also outperforms alignment-based methods such as BlastClust and CD-HIT-EST.

## Methods

A gene is a stretch of DNA that codes for a single polypeptide chain [43]. A gene sequence is a succession of four symbols {A, C, G, T}. Because the similarity between the genes of two species indicates their evolutionary relationship, it is used in many clustering algorithms. The goal of sequence clustering is to partition biological sequences into meaningful/functional groups according to the similarity information, which is calculated using either an alignment-based method or an alignment-free method.

The traditional approach for clustering DNA sequences requires all-by-all comparisons from alignment [44-46]. Given two sequences: *S*_1_ = AGCACACA and *S*_2_ = ACACAGTA, *S*_1_^*P*^ and *S*_2_^*P*^ are used to represent the *p*^th^ characters in *S*_1_ and *S*_2_, respectively. The alignment score [45] for (*S*_1_*S*_2_) is given by

(1) $${\mathrm{Sim}}_{\mathrm{Score}(S_{1},S_{2})}=\sum_{p=1}^{l}E(S_{1}^{p},S_{2}^{p})$$

where *E* is the cost of an alignment operation: deletion, substitution, or insertion. However this distance measure relies on sequence alignment. Since sequence alignment suffers in computational aspect with regard to large biological databases, clustering methods relying on sequence alignment have difficulties in dealing with the large gene data. An alignment-free similarity measure helps avoid the computational complexity of multiple sequence alignment for similarity computation. In this paper we propose a new alignment-free similarity measure, DMk, based on which we developed mBKM to cluster gene sequences.

In the follows, we will present DMk first, and then describe mBKM algorithms.

### A new similarity measure: DMk

In this section, we introduce a new similarity measure which takes into account the occurrence, location and order relation of *k*-tuple in a DNA sequence.

Sequences are numerically transformed to feature vectors that can be processed by data mining algorithms. Let Σ be the alphabet set of nucleotides (Σ = {A, C, G, T}). A sequence of length *s*, *S*, is defined as a linear succession of *s* symbols from Σ. A segment of *k* consecutive symbols in sequence *S* (*k ≤ s*) is designated as a *k*-tuple. There is a set of 4^*k*^ possible *k*-tuples, *W*_*k*_*.* The number of occurrences of a *k*-tuple *w*, *N*_*w*_, is counted by moving a sliding window of length *k* over the sequence with *k* - 1 bp overlapping step size.

To explore the correlation properties of DNA, Nair et al. [47] provided a presentation of genomic data using the inter-nucleotide distance sequence. Based on a similar idea, we utilize the gaps between the locations where *k*-tuple occur in the sequence to explore the sequence structure. For a DNA sequence *Sp*_*r*_ is the location of the *r*^*th*^ occurrence of *k*-tuple *w*, where *p*_0_ = 0. And *α*_*r*_ is given as,

(2) $$\alpha_{r}=\frac{1}{p_{r}-p_{r-1}},1\leq r\leq m$$

in which *m* stands for the number of occurrences of *w*. *α*_*r*_ reflects the density of *w* and is closely related to the location where *w* occurs in the sequence. Each *w* begins at the 1/*α*_1_ position, and {*α*_*1,*_*α*_*2*_*,…,α*_*m*_} for repetition of *w* forms an array whose *r*^*th*^ element indicates the relative position of two neighboring *w* in the sequence. This array allows us to find all subsequent repeats of *w*.

To characterize the order of *α*_*r*_, we define *β*_*j*_ as a partial sum of {*α*_*r*_}. *β*_*j*_ is calculated by the following formula:

(3) $$\beta_{j}=\sum_{r=1}^{j}\alpha_{r},1\leq j\leq m$$

{*α*_*r*_} is a list of non-negative real numbers, and *β*_*j*_ is totally ordered by ≤, so *β*_1_, *β*_2_, …, *β*_*m*_ is also an ordered set. {*α*_1_, *α*_2_,…, *α*_*m*_} and {*β*_1_, *β*_2_,…, *β*_*m*_} determine each other uniquely. *β*_*j*_ is only dependent of the number and positions of *w* and independent on other *k*-tuples. Given the set of {*β*_1_, *β*_2_,…, *β*_*m*_}, one can obtain where *w* occurs and how many times *w* occurs in the sequence.

*Shannon’s entropy*[48], which illuminates the total information measure of source on the average, is a measure of order/disorder. According to [49], when using the totally ordered set {*β*_1_*β*_2_,…, *β*_*m*_} to calculate the probabilities, the Shannon entropy reflects the degree of importance of position in a sequence. We construct a discrete probability distribution $Q=(q_{1},q_{2},\ldots ,q_{m})$, where $q_{i}=\beta_{i}/\sum_{i=1}^{m}\beta_{i}$, and $\sum_{i=1}^{m}q_{i}=1$. The *Shannon entropy* of the discrete probability distribution is calculated by

(4) $$H_{}=-\sum_{i=1}^{m}q_{i}\log_{2}q_{i}$$

For each *k*-tuple *w* in the sequence, not only the information of tuple numbers but also the information of tuple positions is involved in the definition of *H*. We take *H* as the feature of *w* in the sequence, and then construct a vector consisted of *H* of all possible *k*-tuples in the given sequence.

For a fixed *k*, there are 4^*k*^ distinct *k*-tuples to be considered. These *k*-tuples in a fixed 4^*k*^-dimension feature vector are denoted by $\left(H_{1},H_{2},\ldots ,H_{4^{k}}\right)$, where *H*_*i*_ means the feature representation of the *i*th *k*-tuple. This feature vector based on *H* can be regarded as an index for its corresponding sequence.

Cluster analysis algorithms partition objects into groups based on the distances between objects. Euclidean distance is the square root of the summation of the squares of the differences between all pairs of corresponding objects. The *k*-tuple distance is the sum of the differences in frequency over all possible *k*-tuples; on the other hand, we use Euclidean distance between Shannon entropy of *k*-tuples in sequences to measure the similarity. This distance measure method is referred as DMk. For any two sequences *X* and *Y*, DMk can be calculated as:

(5) $$d_{\mathrm{DMk}}(X,Y)=\sqrt{\sum_{i=1}^{4^{k}}{\left(h_{w_{i}}^{X}-h_{w_{i}}^{Y}\right)}^{2}}$$

where $h_{w_{i}}^{X}$ and $h_{w_{i}}^{Y}$ represent the Shannon entropy values of the *i*^*th*^*k*-tuple in sequences *X* and *Y*, respectively. DMk can be calculated from following algorithm:

Algorithm Name: DMk for similarity measure

Input: sequences {*S*_1_, *S*_2_,…, *S*_*N*_}.

Output: similarity matrix, (*d*(*X*,*Y*))_N*N_.

Steps:

1. For each sequence, search and locate each *k*-tuple;

1.1 For each *k*-tuple, use Equation (1) to calculate $\alpha_{r}(1\leq r\leq m)$

1.2 For each *k*-tuple, use Equation (2) to calculate $\beta_{j}(1\leq j\leq m)$;

1.3 For each *k*-tuple, use Equation (3) to calculate *H*;

2. For each sequence, construct 4^*k*^ -component vector by *H* of all *k*-tuples.

3. For any two sequences, use Equation (4) to calculate the distance between the two sequences.

4. Return {*d*}.

### A new clustering algorithm: mBKM

KM can be used to obtain a hierarchical clustering solution using a repeated bisecting approach [50,51]. BKM is such an algorithm and it can produce either a partitional or a hierarchical clustering.

BKM has a linear time complexity in each bisecting step. Recent study [51] concludes BKM outperforms KM as well as the agglomerative approach in terms of accuracy and efficiency. Consequently, the bisecting approach is very attractive in many applications for clustering and genomic data analysis.

BKM initially regards the whole data set as a cluster, and splits one cluster into two subclusters at each bisecting step using KM until singleton clusters are obtained at the leafs or until *K* clusters are obtained. The outcome is structured as a binary tree. There are two key steps in a typical BKM. The first one is the selection of initial centroids. Generally the initial centroids are chosen randomly in BKM. The second key step is the rule, *ζ*, for selection of a existing cluster to be split in each bisecting step. *ζ* is typically given by the following three approaches [50]:

1) Choosing the cluster with largest size;

2) Selecting the cluster with the overall similarity

(6) $$\frac{1}{{\left|C\right|}^{2}}\sum_{\begin{array}{l} s\in C\\ s'\in C \end{array}}d(s,s')$$

The overall similarity is either minimized or maximize, depending on the definition of *d*(*s*, *s*^’^). *C* is a cluster;

3) Using a criterion based on both size and overall similarity.

Because the differences between these methods are small in terms of the final clustering result, the way of splitting the largest remaining cluster is recommended [50].

There are two problems in BKM algorithm:

1. Randomly choosing the initial centroids in BKM may result in too adjacent elements selected. If the initial centroids are too close, the algorithm will reach a local optimization. Moreover, different sets of initial cluster centroids can lead to different final clustering results.

2. The algorithm for choosing one existing cluster to split in each bisecting step usually selects the cluster with the largest size. Although this leads to reasonably good and balanced clustering solution, it cannot gracefully work for datasets where the natural clusters are of different sizes, as it will tend to partition larger clusters first. In real biological data, the number of elements in every cluster may not always be similar.

To address the above two problems and obtain more natural hierarchical solutions, we develop a modified bisecting K-means, mBKM, which choose the initial centroids by the maximum and minimum principle and select the cluster to split based on the compactness of clusters.

1) Selecting Initial Cluster Centroids

In order to achieve stable and reliable clustering results, we use the maximum distance, which can avoid obtaining adjacent elements, to select the initial centroids. For a set of sequences, {*s*_1_, *s*_2_, …, *s*_*N*_}, let *d*(*s*_*i*_, *s*_*j*_)(*i*, *j* = 1, 2, …*N*) be the distance between any two sequences in the dataset. We choose the sequence $s_{c_{1}}$ and $s_{c_{2}}$ as the cluster centroid according the following rule:

(7) $$d_{c_{1},c_{2}}=\max_{i,j=1,2,\ldots ,N}d(s_{i},s_{j})$$

2) Selecting the Cluster to Split

BKM algorithm usually partitions the largest size cluster into two smaller ones and yields clusters with similar size. However, a cluster with large number is not always the loose one. If one existing cluster is a loose one, in which its members are not closely related to each other, the cluster will be selected to be split.

Variance is a measure of how far a set of numbers are spread out from each other, and it can measure the compactness of the clusters. So we select the cluster to split on the basis of the compactness of clusters measured by variance. The variance of cluster *C*_*j*_ is defined as following:

(8) $$\sigma_{j}=\frac{\sum_{s_{i}\in C_{j}}d^{2}(s_{i},\mu_{j})}{n_{j}},1\leq i,j\leq N$$

where *μ*_*j*_ is the centroid of sequences in *C*_*j*_, *d* (*s*_*i*_, *μ*_*j*_) is the distance between *s*_*i*_ and *μ*_*j*_, and *n*_*j*_ is the number of sequences in the cluster.

A small variance of a cluster indicates that the members in the cluster tend to be closely related to the mean. In other words, the smaller the variance is, the more compact the cluster is, and vice versa.

Based on the above idea, we outline mBKM algorithm as follows.

Algorithm Name: mBKM for clustering sequences

Input: sequences {*s*_1_, *s*_2_, …, *s*_*N*_}, a distance function *d* between sequences, the number of clusters *K*.

Output: Set of *K* clusters.

Steps:

1. Initialization: Regard the whole dataset {*s*_1_, *s*_2_, …, *s*_*N*_} as a single cluster.

2. Pick a cluster to split.

3. Find two sub-clusters:

3.1 Select two initial centroids using Equation (6);

3.2 Assign the sequences to the closest centroid;

3.3 Recalculate two centroids based on the sequences assigned to the cluster;

3.4 Repeat steps 3.2 and 3.3 until no change in cluster centroid calculation.

4. Calculate the variance of each cluster according Equation (7) and take the split that produces the clustering result with the highest variance.

5. Repeat steps 2, 3 and 4 until the desired number *K* is reached.

This algorithm outputs a binary tree of sequences, where each leaf represents a sequences and each node represents a sequence collection.

## Results and discussion

The proposed method is evaluated by clustering functionally related gene sequences and by phylogenetic analysis. We present our evaluation results in two parts. The first one aims at testing the efficiency of our similarity measure, DMk. The second one is to illustrate the efficiency of the proposed clustering method, mBKM.

To measure the quality of the clustering results, our experiments adopt F-measure [52] to evaluate the clustering performance. For cluster *j* and class *iF* (*ij*) is defined as:

(9) $$F(i,j)=\frac{2*precision(i,j)*recall(i,j)}{precision(i,j)+recall(i,j)}$$

where *i* =1, 2, …, *ej* = 1, 2, …, *fprecision*(*i*, *j*) = *n*_*ij*_/*n*_*j*_*recall*(*i*, *j*) = *n*_*ij*_/*n*_*i*_*e* is the number of classes, and *f* is the number of clusters. *n*_*ij*_ is the number of the sequences of class *i* in cluster *jn*_*i*_ is the number of the sequences of class *i*, and *n*_*j*_ is the number of the sequences of cluster *j*.

The F-measure of the whole clustering result is defined as:

(10) $$F=\sum_{i}\frac{n_{i}}{N}\max(F(i,j))$$

where *N* is the total number of sequences in the data set. Clearly, an F-measure has a value between 0 and 1. The larger the F-measure is, the better the clustering result is.

### Evaluation of similarity measure

To evaluate the proposed similarity measure, we test DMk on gene sequence data sets and compare it with the *k*-tuple distance. We also verify the effectiveness of DMk by assessing how well it performs on phylogenetic analysis.

#### Gene sequences clustering

Genes of the same family usually share similar sequences, functional domains, and even interacting partners. When a new gene is assigned to a cluster, the biological function of this cluster can be attributed to this gene with high confidence.

Four data sets are extracted from different gene repositories as shown in Table 1. The sequences of DS1 are downloaded from NCBI (http://www.ncbi.nlm.nih.gov). The other three datasets, DS2, DS3 and DS4, are taken from PBIL (http://pbil.univ-lyon1.fr/). DS2 is taken from HOVERGEN of PBIL, a database of homologous vertebrate genes. DS3 is taken from HOGENOM, which contains homologous gene families from microbial organisms. DS4 is randomly selected from HOMOLENS, a database of homologous genes from Ensembl organisms and Ensembl families.

**Table 1:** Description for the Data Sets

**Data**	**Name**	**Number**	**Average length (bp)**	**Description**
DS1	beta-globin	176	1531	Cytochrome P450
	beta-Hemoglobin	89	448	Hemoglobin subunit
	integrin_alpha	142	3360	Integrin, alpha
	ketoacyl-synt1	43	754	Estradiol 17-beta-dehydrogenase 8
	myoglobin	55	478	Cytoglobin Myoglobin
	RWD	93	825	RWD domain-containing protein
	VCL	92	2746	Vinculin
	Histone	81	668	Histone
DS2	HBG106679	22	446	Copper uptake protein 2
	HBG108349	49	718	Prolactin
	HBG079775	26	3152	Transcription elongation factor SPT5
	HBG058842	34	1351	TNFR superfamily member 1A
	HBG002834	92	951	Calumenin/Reticulocalbin
	HBG050441	58	1899	ATP-binding cassette sub-family G member
DS3	HBG093787	32	1769	Hypothetical membrane proteins
	HBG099893	34	430	Putative membrane protein precursor
	HBG415481	65	557	Phasin like/family protein
	HBG423057	32	236	Hypothetical proteins
	HBG050644	99	3129	Beta galactosidase, beta glucuronidase, Evolved beta-D-galactosidase alpha subunit
	HBG364776	48	1069	Formate dehydrogenase gamma subunit precursor
DS4	HBG000080	29	674	BWK-1,CG6617-PA , Zgc:73100 C20orf11 homolog , RH01588p
	HBG060165	28	163	ATP synthase, H + transporting mitochondrial F1 complex/epsilon subunit
	HBG010471	48	1802	Hypothetical Glycosyl transferase, family 25/Endoplasmic reticulum targeting sequence containing protein
	HBG000013	70	318	60 S ribosomal protein L36a-like, 60 S ribosomal protein L42, L44, IP15820p, RPL
	HBG000026	18	3157	Eukaryotic translation initiation factor 2-alpha kinase 3 precursor, Eukaryotic translation initiati
	HBG065748	48	1238	AT20832p,AT27361p, CG10513-PA, CG10514-PA, CG10550-PA, isoform A, CG10553-PA,CG10559-PA,CG10560-P

Four widely used clustering algorithms, including KM, single-linkage clustering (SL), complete-linkage clustering (CL) and average-linkage clustering (AL), have been chosen in the experiments. For comparison, we perform the clustering tests on all data sets using the *k*-tuple distance and DMk distance. In this paper, we set *k* value to 3. For protein coding genes, a tuple size of 3 is a good choice according to reference [22]. We also tested the clustering performance on different *k* values, and the result confirms that a small *k* value is preferred, see Additional file 1: Table S1. For larger *k* values, there are more tuples with zero frequencies and less information is captured by the algorithm.

KM algorithm would yield different results during multiple executions due to its stochastic feature for initialization. We examine KM in ten runs and report the average performance. The AL, CL and SL hierarchical algorithms generate one solution for each of them. We obtain the result of hierarchical clustering algorithms by analyzing the hierarchical tree using the expected number of cluster as input parameters.

According to Table 2, the F-measure values for each of the data sets using DMk are clearly higher than those obtained with the *k*-tuple distance. In our experiments, on average, the value of the F-measure given by DMk is 18% better than by the *k*-tuple distance (p = 0.0165, one-sided paired *t*-test) in KM, 49.7% better in SL (p = 0.0028), 24.9% better in CL (p = 0.016), and 35.8% better in AL (p = 0.01885). Clearly, DMk provides a significant improvement in clustering sequences. On the four data sets, the F-measure of DMk is improved more than 20% compared with that of the *k*-tuple distance during the same clustering process in most cases. DMk outperforms the *k*-tuple distance in the experiments. This is because DMk considers the occurrence, location and order relation of tuples in sequence and can capture more information in the sequence, while the *k*-tuple distance considers frequency alone and ignore the position of tuples in a sequence. In addition, we have tested DMk and *k*-tuple measures on protein sequences with a *k* value of 2, and the results indicate that DMk performs better than *k*-tuple distance (data not shown). Thus in practical DMk measure can also be applied in clustering protein sequences after tuning current algorithm.

**Table 2:** The F-measures of the Data Sets

**Method**	**DS1**	**DS2**	**DS3**	**DS4**
KM with *k*-tuple	0.5738	0.7828	0.5543	0.6532
SL with *k*-tuple	0.3544	0.4148	0.3307	0.3244
CL with *k*-tuple	0.5153	0.7253	0.5588	0.516
AL with *k*-tuple	0.5113	0.6956	0.5578	0.3185
BKM with *k*-tuple	0.5725	0.7876	0.5498	0.6551
mBKM with *k*-tuple	0.5882	0.7913	0.5691	0.6722
KM with DMk	0.7	0.8261	0.7716	0.8284
SL with DMk	0.601	0.7948	0.8188	0.6535
CL with DMk	0.7172	0.9295	0.6868	0.7468
AL with DMk	0.7898	0.9365	0.6963	0.8498
BKM with DMk	0.7346	0.8511	0.8044	0.8813
mBKM with DMk	0.808	0.9645	0.9143	0.9587

#### Phylogenetic analysis

In this experiment, the proposed similarity measure DMk is further tested by phylogenetic analysis. In order to evaluate the similarity measures, we use UPGMA in the PHYLIP package, a widely used clustering algorithm in phylogenetic analysis. The tree is drawn by TREEVIEW program [53].

The selected data set includes the full β-globin gene sequences of 10 species reported by Feng et al. [54], which are downloaded from NCBI (http://www.ncbi.nlm.nih.gov). Their names, accession numbers, locations and lengths are listed in the Additional file 1: Table S2. The similarity/dissimilarity matrices for the full sequences of β-globin gene of the 10 species using DMk are shown in Table 3, respectively. The smaller the distance is, the more similar the two sequences are.

**Table 3:** The similarity/dissimilarity matrix for the 10 full β-globin gene sequences based on DMk

**Species**	**Human**	**Goat**	**Opossum**	**Gallus**	**Lemur**	**Mouse**	**Rat**	**Gorilla**	**Bovine**	**Chimpanzee**
Human	0	22.95	37.65	111.47	14.02	35.21	20.68	3.42	25.07	3.54
Goat		0	41.22	65.70	18.80	35.05	33.93	32.36	6.04	33.05
Opossum			0	42.54	33.29	64.03	51.64	46.35	40.41	49.73
Gallus				0	90.93	80.07	95.26	121.09	61.69	122.65
Lemur					0	21.39	18.50	17.19	18.12	18.74
Mouse						0	16.04	33.64	27.60	37.59
Rat							0	17.69	30.53	20.58
Gorilla								0	33.66	0.80
Bovine									0	35.46
Chimpanzee										0

In Table 3, the most similar species pairs are human-gorilla, human-chimpanzee and gorilla-chimpanzee, which are expected from their evolutionary relationship. A slightly less similar species pair is goat-bovine. On the other hand, gallus is separated from the rest, this coincides with the fact that gallus is the only nonmammalian species among these 10 species. We can also find that opossum is far away from the remaining mammals. These results are consistent with biological morphology.

**Figure 1:**
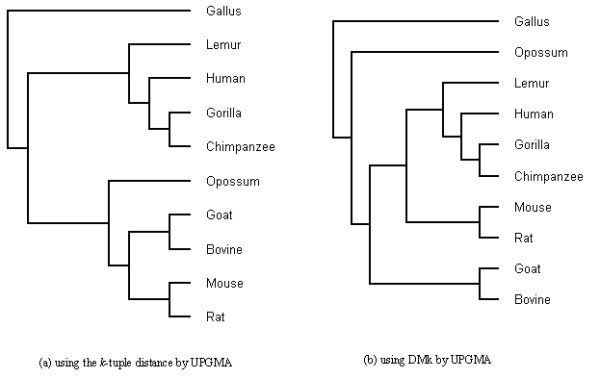
The phylogenetic trees for 10 species using the full DNA sequences of β-globin.

The quality of the constructed tree shows the quality of the distance matrix and the method of abstracting information from DNA sequences. In Figure 1v, we show the phylogenetic tree of 10 β-globin gene sequences based on DMk, generated by UPGMA. For comparison, the phylogenetic tree of the *k*-tuple distance is shown in Figure 1(a).

The tree in Figure 1 (a) has some consistencies with biological morphology. Although it supports the separation of gallus relative to other species, its obvious drawback is that it fails to separate (mouse, rat) and (goat, bovine) from opossum. From Figure 1 (b), gallus is separated from the rest and opossum is far away from the other species. This topology is in good agreement with that presented by Feng et al. [54] and Cao et al. [55] except for the relative position of rodents.

DMk measures the similarity between DNA sequences more effective than the *k*-tuple distance. This is because DMk measures the distance between DNA sequences based on sequence structure and composition. Through evaluation on gene families and constructing phylogenetic trees of full gene sequences of 10 species, we find that DMk gives more competitive results compared to the *k*-tuple distance.

### Evaluation of clustering methods

To evaluate the effectiveness of the proposed clustering algorithm, mBKM, we apply mBKM in clustering gene sequences and compare it with several clustering algorithms. Moreover, we use our method, mBKM with similarity measure DMk, in phylogenetic analysis to show how well the genes are grouped together and how well the resulting trees agree with existing phylogenies.

#### Performance comparison of clustering methods

In order to illustrate the efficiency of mBKM in gene sequence clustering, we ran mBKM with the *k*-tuple distance and DMk on real data sets listed in Table 1. The clustering results are compared with those of KM, SL, CL, AL and BKM algorithms. For BKM, the number of iterations for each bisecting step is set to 5. We ran BKM 10 times to obtain the average F-measure. By combing the six clustering algorithms with two similarity measures, we have 12 combinations of clustering algorithm for performance assessment. The combinations are KM with *k*-tuple, SL with *k*-tuple, CL with *k*-tuple, AL with *k*-tuple, BKM with *k*-tuple, mBKM with *k*-tuple, KM with DMk, SL with DMk, CL with DMk, AL with DMk, BKM with DMk and mBKM with DMk.

The clustering performance of different clustering methods is the result of a combination of factors, including the types of sequence distances used for clustering and the choice of clustering algorithms. Table 2 shows the clustering performance on the data sets for all 12 clustering methods. For each data set, we set the number of cluster as the real number of class during the clustering run. For example, the real number of cluster is 8 in DS1 and 6 in DS2.

From Table 2, we observe that mBKM using DMk achieves best result and clearly outperforms other methods for the four data sets. The average F-measure of mBKM with *k*-tuple is about 2.2% higher than KM with *k*-tuple (p = 0.036), 45% higher than SL with *k*-tuple (p = 0.00195), 11.4% higher than CL with *k*-tuple (p = 0.0424), 19% higher than AL with *k*-tuple (p = 0.08615) and 2.3% higher than BKM (p = 0.0141). For mBKM with DMk, F-measures for DS1, DS2, DS3, and DS4 are 0.808, 0.9645, 0.9143, and 0.9587 respectively. On average, the value of F-measure given by mBKM is 14.2% better than KM (p = 0.00025), 21.3% better than SL (p = 0.0105), 15.4% better than CL (p = 0.02835), 10.1% better in AL (p = 0.0686), and 2.3% higher than BKM (p = 0.0015) respectively. These results show that our method, combining mBKM with DMk, is able to achieve high quality results on all the data sets.

**Figure 2:**
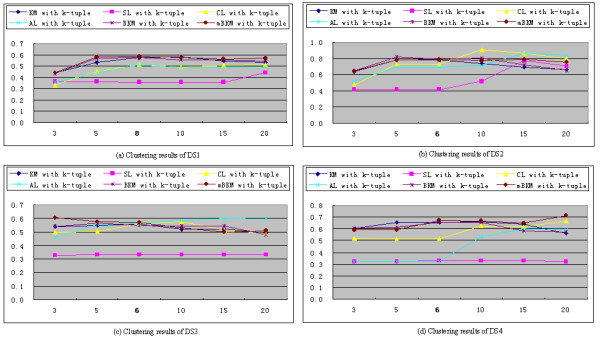
**The distribution of F-measure as a function of the number of clusters based on the *k*-tuple distance (The real numbers of DS1, DS2, DS3 and DS4 are 8, 6, 6, and 6, respectively).**

**Figure 3:**
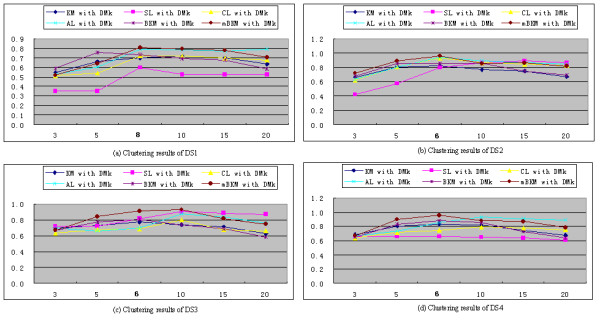
The distribution of F-measure as a function of the number of clusters based on DMk (The real numbers of DS1, DS2, DS3 and DS4 are 8, 6, 6, and 6, respectively).

Because the clustering methods listed in Table 2 use the numbers of cluster as input parameters, we analyze the effects of varying the number of clusters on the clustering performance. This analysis is applied to DS1, DS2, DS3 and DS4 datasets and all 12 combinations. Figures 2 and 3 show the results of these runs based on the *k*-tuple distance and DMk, respectively. The data used for generating these figures are included in Additional file 1: Tables S3-S10.

Figure 2 illustrates the results of the six clustering algorithms with the *k*-tuple distance. From Figure 2 and Additional file 1: Tables S3-S6, mBKM achieves better F-measures than other five clustering algorithms for the real number of clusters on all the data sets. Although the other clustering algorithms give slightly better results in terms of F-measure in some cases, mBKM performs better than the other clustering algorithms in terms of the average of the F-measures values (average values are shown in Additional file 1: Tables S3-S6). This result shows that on average, mBKM performs better than other clustering algorithms for a range of cluster numbers, in the vicinity of real number of clusters. It also implies that varying the number of clusters as input for these clustering algorithms does not affect the performance.

Figure 3 shows the results of clustering algorithms with DMk. mBKM obtains the highest F-measure values among the six clustering algorithms at the real number of clusters. On average, mBKM achieves better results than the other clustering algorithms for DS2, DS3, and DS4. For DS1, the average value of mBKM is very close to that of AL and higher than those of the other clustering algorithms. Overall mBKM produces consistently high quality clusters in the neighborhood of the real number of cluster (data shown in Additional file 1: Tables S7-S10). The F-measures given by mBKM are higher than those of other clustering methods at the corresponding number of clusters in most cases.

From Figures 2 and 3, we can see that DMk achieves better cluster quantity than the *k*-tuple distance in terms of F-measure. Using same clustering algorithm on the same data set, DMk achieves higher average of the F-measure values than the *k*-tuple distance, and DMk also obtains higher F-measures at corresponding number of clusters (data shown in Additional file 1: Tables S3-S10). From both Figures, we find that F-measure changes as the number of cluster changes. As it is known, F-measure is a balanced measure of precision and recall. It is an ideal condition when the number of cluster is equal to the real number. When the number of cluster is greater than or less than the real number, the F-measure will be affected.

With regard to clustering algorithms, SL performs poorly in many cases, and this may be because that SL uses the nearest pair of sequences and may lead to bad splits of one cluster if two or more clusters show different pattern densities. For KM and BKM, the results of many runs are lower than those of mBKM. On the whole, mBKM achieves better results than other clustering algorithms, and mBKM combining with DMk achieves best results among these clustering methods in our experiments.

The task of sequence clustering is to group given sequences into clusters. The similarity measure, DMk, measures the similarity between DNA sequences based solely on the *k*-tuple. It is more effective than the *k*-tuple distance, which is one of the most widely used methods. The clustering algorithm, mBKM, can obtain better clustering results and can reveal the relationships among clusters in hierarchical manner. In the next experiments, we combine mBKM with DMk to clustering DNA sequences.

In order to further illustrate the efficiency of our method, combining mBKM and DMk, we compare mBKM with DMk to two other clustering programs: BlastClust [27] and CD-HIT-EST [39]. BlastClust is an alignment-dependent clustering algorithm. BlastClust is from NCBI Blast package. BlastClust accepts a number of parameters that can be used to control the clustering stringency including thresholds for score density (−*S* parameter), and alignment length (−*L* parameter). CD-HIT-EST is a popular DNA clustering program based on greedy incremental clustering method. CD-HIT-EST groups DNA sequences into clusters that meet a user-defined similarity threshold (−*c* parameter) and uses short-word filters to rapidly determine that if two sequences are similar, which reduces the number of full alignments necessary.

We perform tests using BlastClust and CD-HIT-EST on the data sets listed in Table 1. In order to obtain the best possible performance of BlastClust, we set -*p* as *F* (input type is nucleotide sequence) and vary the input parameters, -*S* and –*L*, to evaluate the results. The score density, –*S* parameter, varies between 10 and 90 with step size 10, and the alignment length, –*L* parameter, varies between 0.1 and 0.9 with step size 0.1. Other parameters are kept default. For CD-HIT-EST, because the sequence identity threshold, -*c* parameter, should be greater than or equal to 0.8 in the program, we vary -*c* parameter between 0.8 and 1 with step size 0.02, and set the word length as default value. The best results from different parameter combination are recorded. For mBKM with DMk, we set the size of *k*-tuple as 3 and use the real number of clusters as input. As BlastClust and CD-HIT-EST do not use the number of clusters as input, we choose the resulting class *i*, which has the max *F(i,j)* for cluster *j*, to calculate the F-measures. The results, which contain the corresponding F-measures and the execution time, are summarized in Table 4.

**Table 4:** **Clustering results on the data sets listed in Table** 1

	**mBKM with DMk**		**BlastClust**		**CD-HIT-EST**	
Data	F-measure	Time(s)	F-measure	Time(s)	F-measure	Time(s)
DS1	0.8080	6.875	0.4525	48	0.2713	39.8
DS2	0.9645	1.844	0.7515	13.6	0.5924	6.4
DS3	0.9143	2.375	0.3693	12.7	0.3157	17.1
DS4	0.9587	1.328	0.5224	9.3	0.4007	6.8

(Time contains the time of similarity measuring and clustering)

Table 4 demonstrates that mBKM with DMk produces good results relative to each original cluster set in terms of F-measure. Every F-measure of mBKM with DMk is higher than 0.8 and the highest is 0.9645. It is also seen in the table that mBKM with DMk outperforms BlastClust and CD-HIT-EST on all the data sets. BlastClust and CD-HIT-EST tend to give more clusters than the real numbers of classes, therefore, BlastClust and CD-HIT-EST give high precision and low recall value. But neither of these two performs well in terms of F-measure. The execution times reported in Table 4 for algorithm comparison show mBKM with DMk is faster than BlastClust and CD-HIT-EST.

For the cases that the real number of clusters is unknown, the performance of our algorithm will be affected. In order to compare with BlastClust and CD-HIT-EST on a relatively fair ground, we can vary the number of clusters and take the average of the F-measure values over the different numbers of clusters. For instance, we run mBKM with DMk with the range of 3–20 numbers and the average values of F-measure are 0.7065, 0.8533, 0.8205 and 0.8429 for DS1, DS2, DS3 and DS4, respectively. As shown in Additional file 1: Tables S7-S10, these values are also higher than the corresponding F-measure of BlastClust and CD-HIT-EST.

#### Phylogenetic analysis

In this experiment, we used mBKM with DMk to construct phylogenetic trees.

1) The clustering result of 10 species

**Figure 4:**
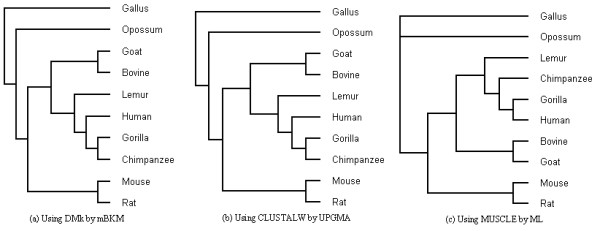
The phylogenetic trees for 10 species using the full DNA sequences of β-globin.

We apply mBKM with DMk to the 10 DNA sequences of β-globin gene in Table 4. The clustering result is shown in Figure 4(a). Using the same data set, we also build the phylogenetic tree using CLUSTALW [56] and MUSCLE [57] for alignment, and UPGMA and Maximum Likelihood (ML) method (in the PHYLIP package) for presenting the tree. Figure 4(b) and 4(c) shows the tree built by CLUSTALW with UPGMA and MUSCLE with ML respectively. The trees built by MUSCLE with UPGMA and CLUSTALW with ML are provided in Figure 1 of Additional file 1.

In Figure 4(a), human, gorilla, chimpanzee and lemur are closer to bovine and goat than to mouse and rat, this topology is in complete agreement with Feng et al. [54] and Cao et al. [55] confirming the outgroup status of *rodents* relative to *ferungulates* and *primates*. Moreover, the tree in Figure 4(a) is identical to the tree in Figure 4(b)4(c) and the tree built MUSCLE with UPGMA. In experiment, the branch (bovine, goat) is not classified well by CLUSTALW with ML. Furthermore, it took about 0.1 second for our method. However, UPGMA with CLUSTALW and MUSCLE for the same data set took 5.1 and 1.2 seconds to build the tree, respectively, and ML with CLUSTALW and MUSCLE took 8 and 4.1 seconds to build the tree, respectively.

2) The Clustering result of 60 H1N1 viruses

**Figure 5:**
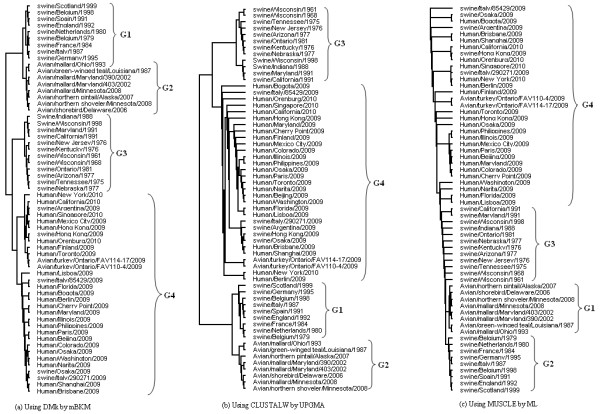
The phylogenetic trees for 60 H1N1 viruses.

H1N1 is subtype of the influenza A virus which can cause illness in humans and many other animal species. Analysis of H1N1 is critical for preparing a strategy to prevent and to control influenza epidemics and pandemics. The H1N1 avian influenza is characterized by its continuous antigen variation, which is mainly caused by the HA and NA proteins in which HA protein has highest rate of mutation. HA protein plays a critical role in identifying and adsorbing the host cell receptor in the infection process, and it is the decisive factor of host specific. We use our method to verify the phylogenetic relationships of H1N1, and the result is included in Additional file 1. The clustering result using mBKM with DMk is shown in Figure 5(a). As a comparison, we also use CLUSTALW with UPGMA and MUSCLE with ML to construct the phylogenetic tree and they are presented in Figure 5(b) and 5(c).

As is seen from Figure 5(a), 60 H1N1 viruses are distinctly divided into four main groups using our method. The four groups, include European swine older than 2009 (G1), the avian older than 2009 (G2), American swine older than 2009 (G3) and the new 2009 viruses from human, swine and avian (G4). The result shows that the new 2009 human H1N1 viruses have closer relationship with old American swine than old avian and European swine. This grouping result is generally consistent with the topology given by CLUSTALW with UPGMA, which is shown in Figure 5(b), and the one presented by MUSCLE with UPGMA, which is provided in the Additional file 1, as well as the result suggested by zhao et al. [58]. Figure 5(c), built by MUSCLE using ML method, also shows the new 2009 human H1N1 viruses have close relationship with old American swine except the position of the group (old avian swine, European swine) is different from the positions in Figure 5(a) and 5(b). CLUSTALW with ML (in Additional file 1) also classifies the 60 H1N1 viruses into four groups except that swine/Wisconsin/1961 and swine/Wisconsin/1961 are not classified well.

Our method analyzed the 60 H1N1 viruses within 1 second, while UPGMA with CLUSTALW and MUSCLE of the same data set took 460 and 60.1 seconds to build the tree, and ML with CLUSTALW and MUSCLE took 571 and 188.1 seconds to build the tree, respectively.

Our method, mBKM with DMk, performs well when clustering 10 species and 60 H1N1 viruses. It obtains similar results to the alignment-based method. Furthermore, our method is much faster than the alignment-based methods.

**Figure 6:**
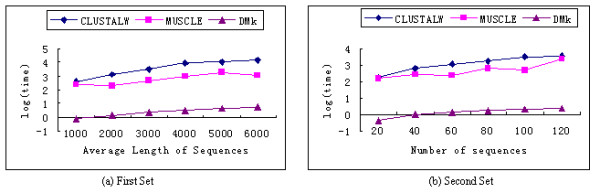
The time comparison of three methods.

In order to compare the speed of our method with the multiple sequence alignment based methods, CLUSTALW and MUSCLE, we performed the test on two sets of sequences. The first set consists of six datasets. All the six datasets include 100 sequences. The lengths of all sequences in the six datasets are around 1000, 2000, 3000, 4000, 5000 and 6000 respectively. Another set also consists of six datasets. The number of sequences in each dataset is 20, 40, 60, 80, 100, 120 respectively; the lengths of all the sequences are around 3000. Because ML method is slower than UPGMA, we use UPGMA to build the phylogenetic tree of the results from CLUSTALW and MUSCLE and record the time used for each method. The results in Figure 6 show that our method is much faster than the other two methods. The actual time differences are much higher than the visual differences in the figure since we are using the log(time) as the label of y-axis.

#### Scalability test

For DMk, the time complexity of transforming the gene sequence *s*_1_⋯*s*_*l*_ to a vector is *O* (*l*4^*K*^), thus the time complexity of generating the vectors for the whole sequence database is $O(N\bar{l}4^{k})$, where $\bar{l}$ is the average length of the sequences and *N* is the number of sequences. The value of *k* set to 3 yields good results in our experiments, and we fix *k* to 3 as the size of *k*-tuple. DMk have linear time complexity with respect to both $\bar{l}$ and *N*.

The time consumed for mBKM calculation is primarily determined by choosing the initial cluster centroids. For *N* sequences, this step has a time complexity of *O* (*N*^2^). The time complexity of clustering step in mBKM is *O* (*N* log *K*). The following scalability test on our method, mBKM with DMk, confirms that our method has linear time complexity with respect to the average length of the sequences. The scalability test uses theoretical model sequences composed of the four symbols ‘A’, ‘C’, ’G’ and ‘T’. The method is implemented in Java and on a computer with 3.00 GHz CPU and 2 GB RAM.

**Figure 7:**
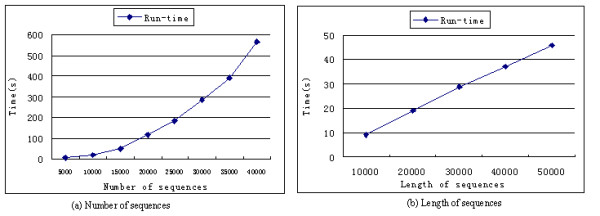
The relationship between the runtime and different numbers of sequences and length of sequences.

Figure 7(a) illustrates the relationships between the runtime and the number of sequences (implemented on a computer with 8 GB RAM). To test the scalability with respect to the number of sequences, we use five data sets which consist of 5000, 10000, 15000, 20000, 25000, 30000, 35000 and 40000 sequences. Each data set contains 10 clusters and all the sequences have the same length, 100. The curve in Figure 7(a) is primarily consistent with the time complexity of mBKM with *O* (*N*^2^). The scalability with respect to the length of sequences was tested on five datasets with five different sequence lengths: 10000, 20000, 30000, 40000, 50000 and each set consists of 4 clusters and 100 sequences. The sensitivity with respect to the length of the sequence is illustrated in Figure 7(b), from which we can see that the time of our method increases linearly when the length of sequences increases.

## Conclusions

In this paper, we presented a novel approach for DNA sequence clustering, mBKM, based on a new sequence similarity measure, DMk, which is extracted from DNA sequences based on the position and composition of oligonucleotide pattern. The experimental results show the method of combining mBKM with DMk is effective in classifying DNA sequences with similar biological characteristics and in discovering the underlying relationship among the sequences. In addition, DMk can achieve comparable or better accuracy than the frequency-based distance measure. Our proposed method can be applied to study gene families and it can also help with the prediction of novel genes. Furthermore, mBKM with DMk can generate cluster trees that are useful to understand the processes governing the gene evolution. In addition, our method may be extended for protein sequence analysis and metagenomics of identifying source organisms of metagenmic data. Our method has limitations too. For example, the method did not consider edge length, and has not address problems with long repeated sequences or long insertions. In future we will try to address these problems.

## Competing interests

The authors declare that there are no competing interests.

## Authors’ contributions

DW designed the algorithm, conducted the experiments, and wrote the manuscript. QJ supervised the project and proposed data mining algorithm. YW guided the experiments, wrote the manuscript and analyzed the results. SW guided the experiment analysis, and proposed ideas for sequence clustering algorithm. All authors read and approved the final manuscript.

## Supplementary Material

Additional file 1 Supplementary Data. Click here for file
