# Supplementary material for: A novel hierarchical clustering algorithm for gene sequences
Source: BMC Bioinformatics. 2012 Jul 23;13:174. doi: 10.1186/1471-2105-13-174 (PMC3443659; doi:10.1186/1471-2105-13-174)
Supplement: Additional file 1 — Supplementary Data. [file 1471-2105-13-174-S1.doc]

Supplementary Data:

A Novel Hierarchical Clustering Algorithm for Gene Sequences

Dan Wei, Qingshan Jiang, Yanjie Wei, Shengrui Wang

Table 1. The F-measure of different clustering algorithms with k=2, 3, 4, 5, 6 on DS1, DS2, DS3, and DS4

| **DS1** | k=2 | | k=3 | | k=4 | | k=5 | | k=6 | |
| --- | --- | --- | --- | --- | --- | --- | --- | --- | --- | --- |
|  | k-tuple | DMk | k-tuple | DMk | k-tuple | DMk | k-tuple | DMk | k-tuple | DMk |
| KM | 0.5612 | 0.7226 | 0.5738 | 0.7000 | 0.6067 | 0.7009 | 0.5829 | 0.6292 | 0.4875 | 0.5073 |
| SL | 0.3543 | 0.6821 | 0.3544 | 0.6010 | 0.3614 | 0.5989 | 0.2533 | 0.2525 | 0.2534 | 0.2520 |
| CL | 0.4734 | 0.7602 | 0.5153 | 0.7172 | 0.5308 | 0.6227 | 0.4735 | 0.4089 | 0.4351 | 0.3367 |
| AL | 0.5049 | 0.7588 | 0.5113 | 0.7898 | 0.4485 | 0.6422 | 0.3567 | 0.4329 | 0.3516 | 0.2488 |
| **DS2** | k=2 | | k=3 | | k=4 | | k=5 | | k=6 | |
|  | k-tuple | DMk | k-tuple | DMk | k-tuple | DMk | k-tuple | DMk | k-tuple | DMk |
| KM | 0.7746 | 0.8437 | 0.7828 | 0.8261 | 0.7514 | 0.7980 | 0.6938 | 0.7697 | 0.5508 | 0.6153 |
| SL | 0.6102 | 0.6910 | 0.4148 | 0.7948 | 0.3998 | 0.8395 | 0.3998 | 0.4318 | 0.3998 | 0.4057 |
| CL | 0.6707 | 0.9295 | 0.7253 | 0.9295 | 0.7026 | 0.8206 | 0.5729 | 0.5953 | 0.5247 | 0.4057 |
| AL | 0.7316 | 0.9446 | 0.6956 | 0.9365 | 0.7107 | 0.8743 | 0.5692 | 0.5953 | 0.3996 | 0.4057 |
| **DS3** | k=2 | | k=3 | | k=4 | | k=5 | | k=6 | |
|  | k-tuple | DMk | k-tuple | DMk | k-tuple | DMk | k-tuple | DMk | k-tuple | DMk |
| KM | 0.5302 | 0.8051 | 0.5543 | 0.7716 | 0.5876 | 0.7952 | 0.5702 | 0.6111 | 0.5250 | 0.5567 |
| SL | 0.3300 | 0.7117 | 0.3307 | 0.8188 | 0.3310 | 0.7134 | 0.3304 | 0.5169 | 0.3307 | 0.3256 |
| CL | 0.5406 | 0.7744 | 0.5588 | 0.6868 | 0.5494 | 0.6606 | 0.5656 | 0.4312 | 0.3815 | 0.3377 |
| AL | 0.5737 | 0.8098 | 0.5578 | 0.6963 | 0.3817 | 0.6594 | 0.3310 | 0.4869 | 0.3260 | 0.3145 |
| **DS4** | k=2 | | k=3 | | k=4 | | k=5 | | k=6 | |
|  | k-tuple | DMk | k-tuple | DMk | k-tuple | DMk | k-tuple | DMk | k-tuple | DMk |
| KM | 0.6225 | 0.8632 | 0.6521 | 0.8284 | 0.6792 | 0.8241 | 0.6130 | 0.7721 | 0.5641 | 0.5333 |
| SL | 0.3232 | 0.6577 | 0.3244 | 0.6535 | 0.3250 | 0.4071 | 0.3247 | 0.3962 | 0.3247 | 0.3346 |
| CL | 0.5132 | 0.8780 | 0.5160 | 0.7468 | 0.6830 | 0.7253 | 0.3977 | 0.3791 | 0.4033 | 0.3729 |
| AL | 0.4403 | 0.7413 | 0.3185 | 0.8498 | 0.3965 | 0.7297 | 0.3985 | 0.3792 | 0.3913 | 0.3490 |

Table 2. The full DNA sequences of β-globin gene of 10 species

| **No.** | **Species** | **Accession** | **Location** | **Length (nt)** |
| --- | --- | --- | --- | --- |
| 1 | Human | U01317 | 62187–63610 | 1424 |
| 2 | Goat | M15387 | 279-1749 | 1471 |
| 3 | Opossum | J03643 | 467-2488 | 2022 |
| 4 | Gallus | V00409 | 465-1810 | 1346 |
| 5 | Lemur | M15734 | 154-1595 | 1442 |
| 6 | Mouse | V00722 | 275-1462 | 1188 |
| 7 | Rat | X06701 | 310-1505 | 1196 |
| 8 | Gorilla | X61109 | 4538-5881 | 1344 |
| 9 | Bovine | X00376 | 278-1741 | 1464 |
| 10 | Chimpanzee | X02345 | 4189-5532 | 1344 |

Table 3. The F-measure values of DS1 over the different numbers of clusters on the *k*-tuple distance

|  | 3 | 5 | **8** | 10 | 15 | 20 | Average |
| --- | --- | --- | --- | --- | --- | --- | --- |
| KM with k-tuple | 0.4414 | 0.5300 | 0.5738 | 0.5787 | 0.5458 | 0.5297 | 0.5332 |
| SL with k-tuple | 0.3610 | 0.3612 | 0.3544 | 0.3545 | 0.3564 | 0.4382 | 0.3709 |
| CL with k-tuple | 0.3340 | 0.4507 | 0.5153 | 0.4900 | 0.5088 | 0.5152 | 0.4690 |
| AL with k-tuple | 0.3540 | 0.4410 | 0.5113 | 0.4819 | 0.4902 | 0.4922 | 0.4618 |
| BKM with k-tuple | 0.4323 | 0.5677 | 0.5725 | 0.5561 | 0.5548 | 0.5457 | 0.5382 |
| mBKM with k-tuple | 0.4416 | 0.5767 | 0.5882 | 0.5732 | 0.5628 | 0.5655 | **0.5513** |

Table 4. The F-measure values of DS2 over the different numbers of clusters on the *k*-tuple distance

|  | 3 | 5 | **6** | 10 | 15 | 20 | Average |
| --- | --- | --- | --- | --- | --- | --- | --- |
| KM with k-tuple | 0.6327 | 0.7791 | 0.7828 | 0.7388 | 0.6938 | 0.6641 | 0.7152 |
| SL with k-tuple | 0.4148 | 0.4156 | 0.4148 | 0.5203 | 0.7706 | 0.7067 | 0.5405 |
| CL with k-tuple | 0.4767 | 0.7253 | 0.7253 | 0.9085 | 0.8659 | 0.7972 | 0.7498 |
| AL with k-tuple | 0.5148 | 0.6973 | 0.6956 | 0.7156 | 0.8630 | 0.8398 | 0.7210 |
| BKM with k-tuple | 0.6420 | 0.8186 | 0.7876 | 0.8083 | 0.7179 | 0.6556 | 0.7383 |
| mBKM with k-tuple | 0.6410 | 0.7755 | 0.7913 | 0.7713 | 0.7877 | 0.7500 | **0.7528** |

Table 5. The F-measure values of DS3 over the different numbers of clusters on the *k*-tuple distance

|  | 3 | 5 | **6** | 10 | 15 | 20 | Average |
| --- | --- | --- | --- | --- | --- | --- | --- |
| KM with k-tuple | 0.5387 | 0.5472 | 0.5543 | 0.5205 | 0.5194 | 0.4919 | 0.5287 |
| SL with k-tuple | 0.3296 | 0.3302 | 0.3307 | 0.3323 | 0.3337 | 0.3348 | 0.3319 |
| CL with k-tuple | 0.4983 | 0.5048 | 0.5588 | 0.5740 | 0.5153 | 0.4890 | 0.5234 |
| AL with k-tuple | 0.4578 | 0.5530 | 0.5578 | 0.5862 | 0.6004 | 0.5936 | **0.5581** |
| BKM with k-tuple | 0.5383 | 0.5649 | 0.5498 | 0.5417 | 0.5415 | 0.4794 | 0.5360 |
| mBKM with k-tuple | 0.6026 | 0.5750 | 0.5691 | 0.5285 | 0.4995 | 0.5106 | 0.5476 |

Table 6. The F-measure values of DS4 over the different numbers of clusters on the *k*-tuple distance

|  | 3 | 5 | **6** | 10 | 15 | 20 | Average |
| --- | --- | --- | --- | --- | --- | --- | --- |
| KM with k-tuple | 0.6005 | 0.6532 | 0.6521 | 0.6601 | 0.6338 | 0.5595 | 0.6265 |
| SL with k-tuple | 0.3240 | 0.3234 | 0.3244 | 0.3241 | 0.3254 | 0.3221 | 0.3239 |
| CL with k-tuple | 0.5221 | 0.5145 | 0.5160 | 0.6243 | 0.6196 | 0.6662 | 0.5771 |
| AL with k-tuple | 0.3227 | 0.3232 | 0.3185 | 0.5306 | 0.5985 | 0.5922 | 0.4476 |
| BKM with k-tuple | 0.6089 | 0.6131 | 0.6551 | 0.6559 | 0.5813 | 0.5731 | 0.6146 |
| mBKM with k-tuple | 0.5917 | 0.5922 | 0.6722 | 0.6672 | 0.6508 | 0.7125 | **0.6478** |

Table 7. The F-measure values of DS1 over the different numbers of clusters on the DMk

|  | 3 | 5 | 8 | 10 | 15 | 20 | Average |
| --- | --- | --- | --- | --- | --- | --- | --- |
| KM with DMk | 0.5472 | 0.6607 | 0.7000 | 0.7179 | 0.6968 | 0.6333 | 0.6593 |
| SL with DMk | 0.3509 | 0.3541 | 0.6010 | 0.5272 | 0.5269 | 0.5263 | 0.4811 |
| CL with DMk | 0.5126 | 0.5404 | 0.7172 | 0.7209 | 0.6993 | 0.6748 | 0.6442 |
| AL with DMk | 0.5237 | 0.6050 | 0.7898 | 0.7853 | 0.7689 | 0.7891 | **0.7103** |
| BKM with DMk | 0.5927 | 0.7556 | 0.7346 | 0.6887 | 0.6742 | 0.5848 | 0.6717 |
| mBKM with DMk | 0.5180 | 0.6411 | 0.8080 | 0.7919 | 0.7756 | 0.7045 | 0.7065 |

Table 8. The F-measure values of DS2 over the different numbers of clusters on the DMk

|  | 3 | 5 | 6 | 10 | 15 | 20 | Average |
| --- | --- | --- | --- | --- | --- | --- | --- |
| KM with DMk | 0.6637 | 0.8019 | 0.8261 | 0.7678 | 0.7506 | 0.6724 | 0.7471 |
| SL with DMk | 0.4257 | 0.5806 | 0.7948 | 0.8680 | 0.8906 | 0.8655 | 0.7376 |
| CL with DMk | 0.6267 | 0.8025 | 0.9295 | 0.8660 | 0.8438 | 0.8221 | 0.8151 |
| AL with DMk | 0.6289 | 0.8096 | 0.9365 | 0.8935 | 0.8611 | 0.8440 | 0.8289 |
| BKM with DMk | 0.6842 | 0.8561 | 0.8511 | 0.8552 | 0.7470 | 0.6886 | 0.7804 |
| mBKM with DMk | 0.7225 | 0.8889 | 0.9645 | 0.8589 | 0.8644 | 0.8207 | **0.8533** |

Table 9. The F-measure values of DS3 over the different numbers of clusters on the DMk

|  | 3 | 5 | 6 | 10 | 15 | 20 | Average |
| --- | --- | --- | --- | --- | --- | --- | --- |
| KM with DMk | 0.6990 | 0.7311 | 0.7716 | 0.7411 | 0.7146 | 0.6252 | 0.7138 |
| SL with DMk | 0.7193 | 0.7188 | 0.8188 | 0.9009 | 0.8857 | 0.8686 | 0.8187 |
| CL with DMk | 0.6313 | 0.6800 | 0.6868 | 0.7996 | 0.6819 | 0.6576 | 0.6895 |
| AL with DMk | 0.6920 | 0.6570 | 0.6963 | 0.8790 | 0.8315 | 0.7544 | 0.7517 |
| BKM with DMk | 0.6679 | 0.7775 | 0.8045 | 0.7405 | 0.6849 | 0.5818 | 0.7095 |
| mBKM with DMk | 0.6656 | 0.8434 | 0.9143 | 0.9327 | 0.8150 | 0.7518 | **0.8205** |

Table 10. The F-measure values of DS4 over the different numbers of clusters on the DMk

|  | 3 | 5 | 6 | 10 | 15 | 20 | Average |
| --- | --- | --- | --- | --- | --- | --- | --- |
| KM with DMk | 0.6864 | 0.7997 | 0.8284 | 0.8221 | 0.7471 | 0.6751 | 0.7598 |
| SL with DMk | 0.6548 | 0.6599 | 0.6535 | 0.6466 | 0.6415 | 0.6130 | 0.6449 |
| CL with DMk | 0.6369 | 0.7120 | 0.7468 | 0.7912 | 0.7781 | 0.7565 | 0.7369 |
| AL with DMk | 0.6675 | 0.7368 | 0.8498 | 0.9285 | 0.9087 | 0.8929 | 0.8307 |
| BKM with DMk | 0.6531 | 0.8294 | 0.8813 | 0.8543 | 0.7250 | 0.6390 | 0.7637 |
| mBKM with DMk | 0.6630 | 0.8955 | 0.9587 | 0.8808 | 0.8712 | 0.7884 | **0.8429** |

Table 11. The HA gene sequences of 60 H1N1 viruses

| **No.** | **Abbreviation** | **Accession** | **Length (bp)** |
| --- | --- | --- | --- |
| 1 | swine/Wisconsin/1998 | AF222034 | 1773 |
| 2 | swine/Belgium/1998 | FJ805962 | 1745 |
| 3 | swine/Scotland/1999 | CY037960 | 1698 |
| 4 | swine/Argentina/2009 | CY044256 | 1731 |
| 5 | swine/Osaka/2009 | AB531444 | 1701 |
| 6 | swine/Italy/290271/2009 | CY053619 | 1701 |
| 7 | swine/Hong Kong/2009 | CY061765 | 1749 |
| 8 | swine/Italy/85429/2009 | CY057078 | 1701 |
| 9 | swine/Belgium/1979 | CY037898 | 1698 |
| 10 | swine/Netherlands/1980 | AF091314 | 1778 |
| 11 | swine/France/1984 | CY037975 | 1698 |
| 12 | swine/Italy/1987 | AF091315 | 1777 |
| 13 | swine/Spain/1991 | CY037999 | 1698 |
| 14 | swine/England/1992 | CY038007 | 1698 |
| 15 | swine/Germany/1995 | AM920728 | 1777 |
| 16 | swine/Wisconsin/1961 | AF091307 | 1778 |
| 17 | swine/Wisconsin/1968 | EU139825 | 1701 |
| 18 | swine/Tennessee/1975 | CY022397 | 1744 |
| 19 | swine/New Jersey/1976 | K00992 | 1778 |
| 20 | swine/Kentucky/1976 | CY022349 | 1744 |
| 21 | swine/Nebraska/1977 | CY022373 | 1744 |
| 22 | swine/Arizona/1977 | CY025002 | 1744 |
| 23 | swine/Ontario/1981 | CY022381 | 1744 |
| 24 | swine/Indiana/1988 | M81707 | 1778 |
| 25 | swine/Maryland/1991 | CY022477 | 1744 |
| 26 | swine/California/1991 | CY028780 | 1732 |
| 27 | Avian/mallard/Ohio/1993 | CY018885 | 1743 |
| 28 | Avian/mallard/Maryland/390/2002 | EU026074 | 1773 |
| 29 | Avian/mallard/Maryland/403/2002 | EU026082 | 1771 |
| 30 | Avian/mallard/Minnesota/2008 | CY042328 | 1607 |
| 31 | Avian/northern shoveler/Minnesota/2008 | CY042776 | 1735 |
| 32 | Avian/shorebird/Delaware/2006 | CY043920 | 1701 |
| 33 | Avian/northern pintail/Alaska/2007 | GU168292 | 1651 |
| 34 | Avian/green-winged teal/Louisiana/1987 | GU050711 | 1751 |
| 35 | Avian/turkey/Ontario/FAV114-17/2009 | HM370975 | 1777 |
| 36 | Avian/turkey/Ontario/FAV110-4/2009 | HM370967 | 1777 |
| 37 | Human/Cherry Point/2009 | CY049828 | 1698 |
| 38 | Human/Bogota/2009 | CY049836 | 1698 |
| 39 | Human/Toronto/2009 | FJ974026 | 1773 |
| 40 | Human/Illinois/2009 | GQ323530 | 1701 |
| 41 | Human/Colorado/2009 | GQ221812 | 1701 |
| 42 | Human/Finland/2009 | GQ283488 | 1743 |
| 43 | Human/Philippines/2009 | GQ243753 | 1687 |
| 44 | Human/Shanghai/2009 | GQ225357 | 1701 |
| 45 | Human/Osaka/2009 | GQ219578 | 1701 |
| 46 | Human/Paris/2009 | GQ214138 | 1720 |
| 47 | Human/Beijing/2009 | GQ183617 | 1728 |
| 48 | Human/New York/2010 | CY064995 | 1734 |
| 49 | Human/California/2010 | CY064987 | 1734 |
| 50 | Human/Orenburg/2010 | HM569740 | 1744 |
| 51 | Human/Mexico City/2009 | CY064756 | 1734 |
| 52 | Human/Singapore/2010 | CY063835 | 1740 |
| 53 | Human/Berlin/2009 | CY064772 | 1721 |
| 54 | Human/Florida/2009 | GQ160526 | 1701 |
| 55 | Human/Brisbane/2009 | GQ160611 | 1687 |
| 56 | Human/Maryland/2009 | GQ160565 | 1701 |
| 57 | Human/Washington/2009 | GQ160578 | 1701 |
| 58 | Human/Narita/2009 | GQ165814 | 1701 |
| 59 | Human/Lisboa/2009 | GQ166752 | 1677 |
| 60 | Human/Hong Kong/2009 | GQ168606 | 1742 |


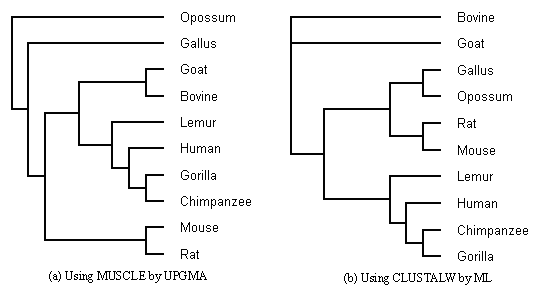


Figure 1. The phylogenetic trees for 10 species using the full DNA sequences of β-globin


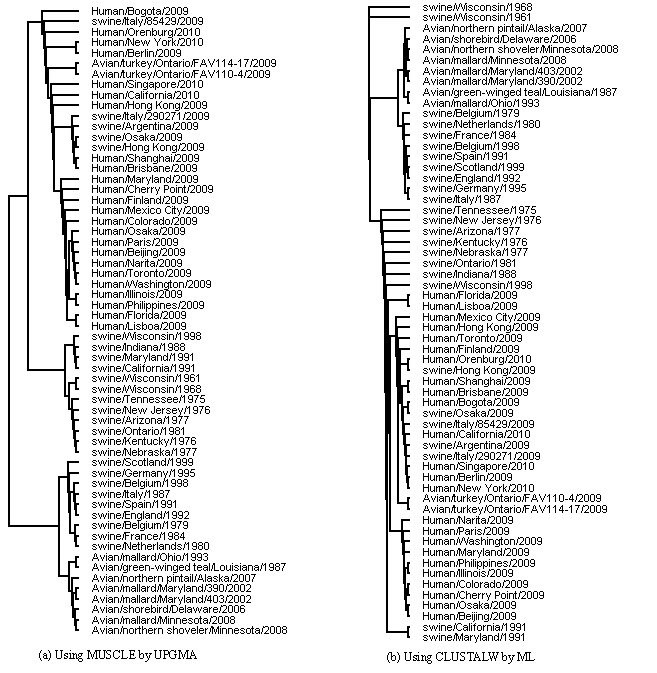


Figure 2. The phylogenetic trees for 60 H1N1 viruses
